# Successful radiotherapy for endometrial serous carcinoma with local repeated recurrence

**DOI:** 10.1007/s13691-018-0323-4

**Published:** 2018-04-05

**Authors:** Shinya Kusumoto, Hisaya Fujiwara, Maiko Sagawa, Takahiro Nobuzane, Toshihiro Nishida, Yukio Akagi, Yutaka Hirokawa, Yasuhiro Katsube

**Affiliations:** 1 Department of Obstetrics and Gynecology, Chugoku Rosai Hospital, 1-5-1 Hiro Tagaya, Kure, Hiroshima 737-0193 Japan; 2 Department of Pathology, Chugoku Rosai Hospital, 1-5-1 Hiro Tagaya, Kure, Hiroshima 737-0193 Japan; 3 Hiroshima Heiwa Clinic, State of the Art Treatment Center, 1-31 Kawara-machi, Naka-ku, Hiroshima, 730-0856 Japan

**Keywords:** Endometrial serous carcinoma, Multiple recurrence, Intensity modulated radiation therapy, Complete remission

## Abstract

The incidence of endometrial serous carcinoma (ESC) has been increasing, and ESC is resistant to treatment. We report a patient with ESC who responded to radiotherapy for multiple recurrences. The first recurrence was detected in the vaginal wall and left internal iliac lymph node 5 months after the initial treatment. Concurrent chemoradiotherapy (CCRT) was administered. Radiation was delivered using the intensity modulated radiation therapy technique. The second recurrent tumor was detected in the right internal iliac lymph node after 4 months, and CCRT was conducted. After 4 months, the third recurrence was detected in the right common iliac node, and CCRT was performed. After 8 months, the fourth recurrence was detected in the horizontal portion of the duodenum, and radiotherapy was administered. After 9 months, the fifth recurrence was detected in the vaginal wall. Interstitial brachytherapy was conducted. Grade 2 gastrointestinal injury, nausea and radiodermatitis were observed. During the subsequent 13-month follow-up, there has been no recurrence. Although ESC is resistant to treatment, radiotherapy could be effective in some cases. Even when multiple recurrences occur, radiotherapy may be considered a treatment option if the irradiation level is permissible.

## Introduction

In Japan, the incidence of endometrial serous carcinoma (ESC) has been increasing [1]. ESC is a histological subtype of endometrial carcinoma with a poor prognosis [2]. The recurrence of ESC frequently occurs and its treatment is very difficult. In this study, we report a 68-year-old woman with ESC who experienced repeated recurrence 5 times and responded to radiotherapy for each recurrence.

## Case report

**Fig. 1:**
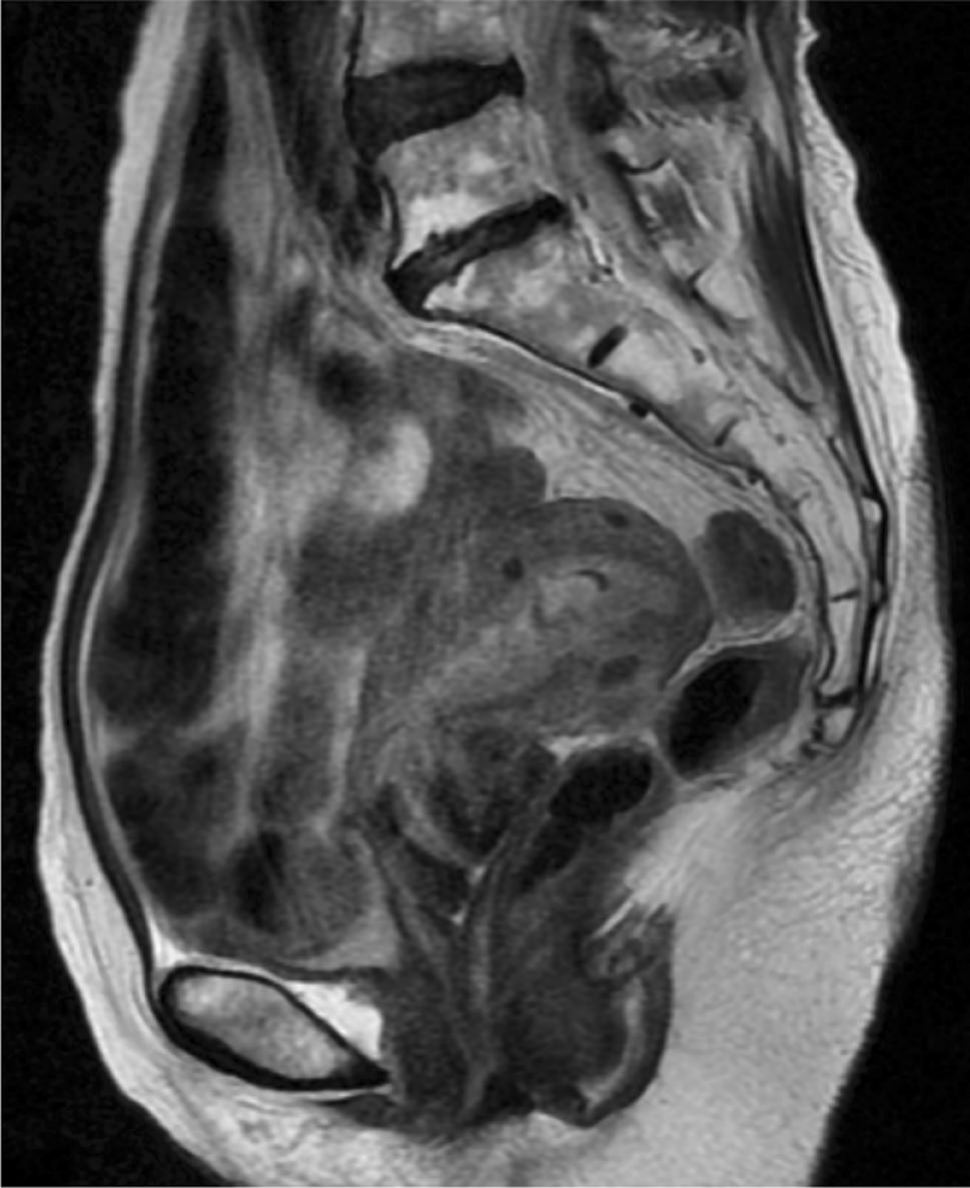
Sagittal T2-weighted MRI findings. The thickened and uneven endometrium lining was observed. *MRI* magnetic resonance imaging

**Fig. 2:**
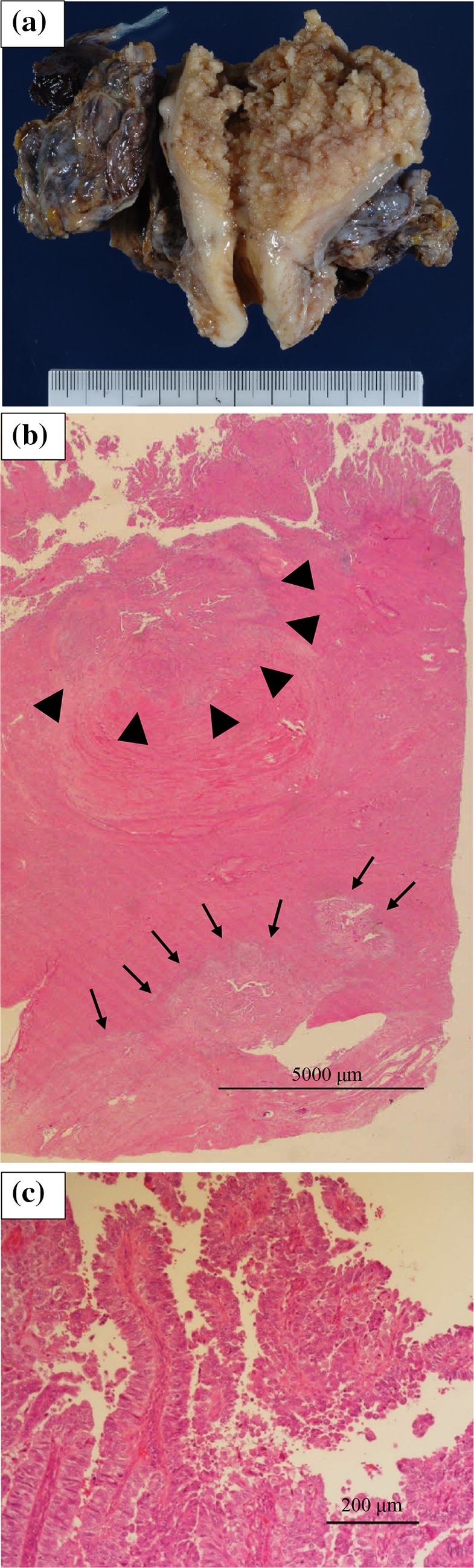
**a** The size of the resected tumor was 60 mm. **b** The cancer had spread into the inner half of the myometrium (triangles), and perimetrium metastases were observed (arrows). **c** The histologic type was endometrial serous carcinoma

**Fig. 3:**
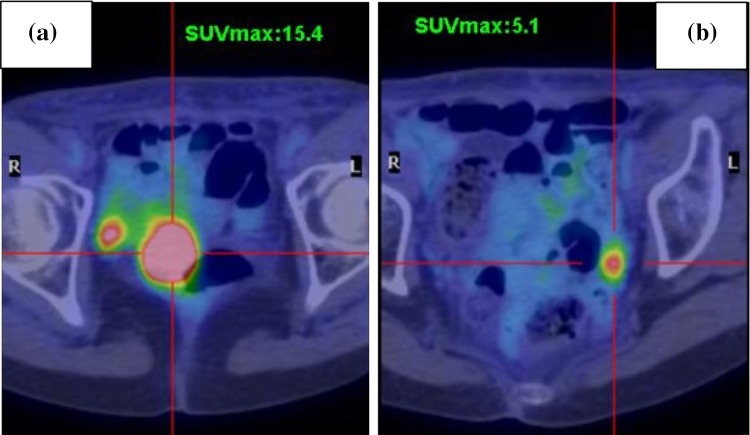
FDG-PET shows accumulation at the vaginal wall (**a**) and left internal iliac lymph node (**b**). *FDG-PET* fluorodeoxyglucose positron emission tomography

**Fig. 4:**
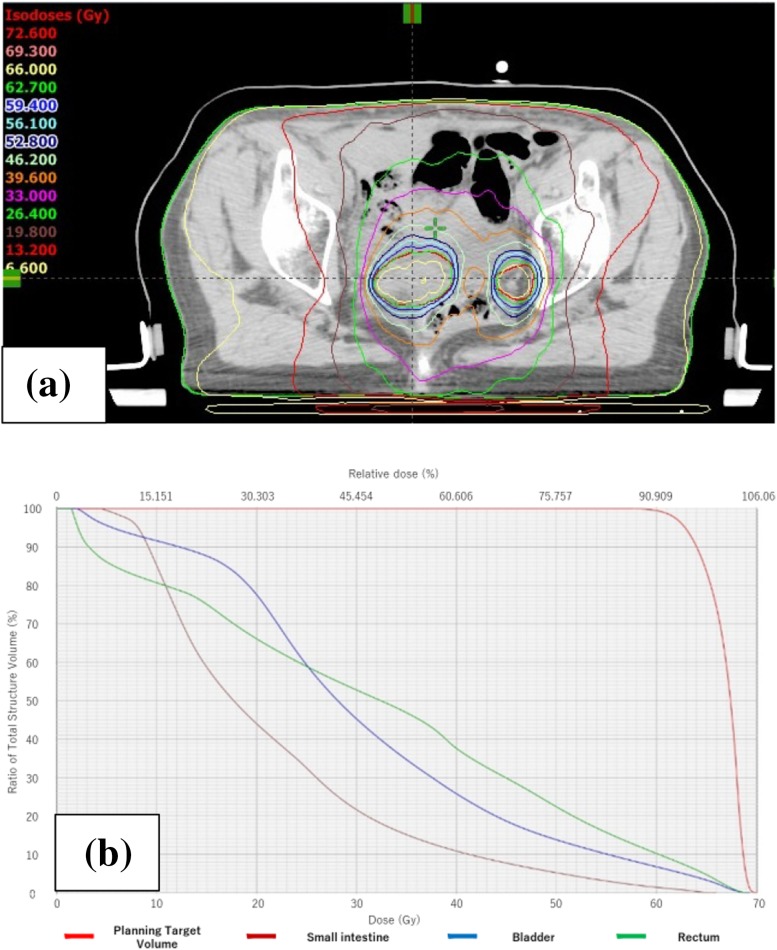
**a** Dose distribution of first IMRT. **b** Dose volume histogram of the small intestine, rectum, bladder and planning target volume. *IMRT* intensity modulated radiation therapy

**Fig. 5:**
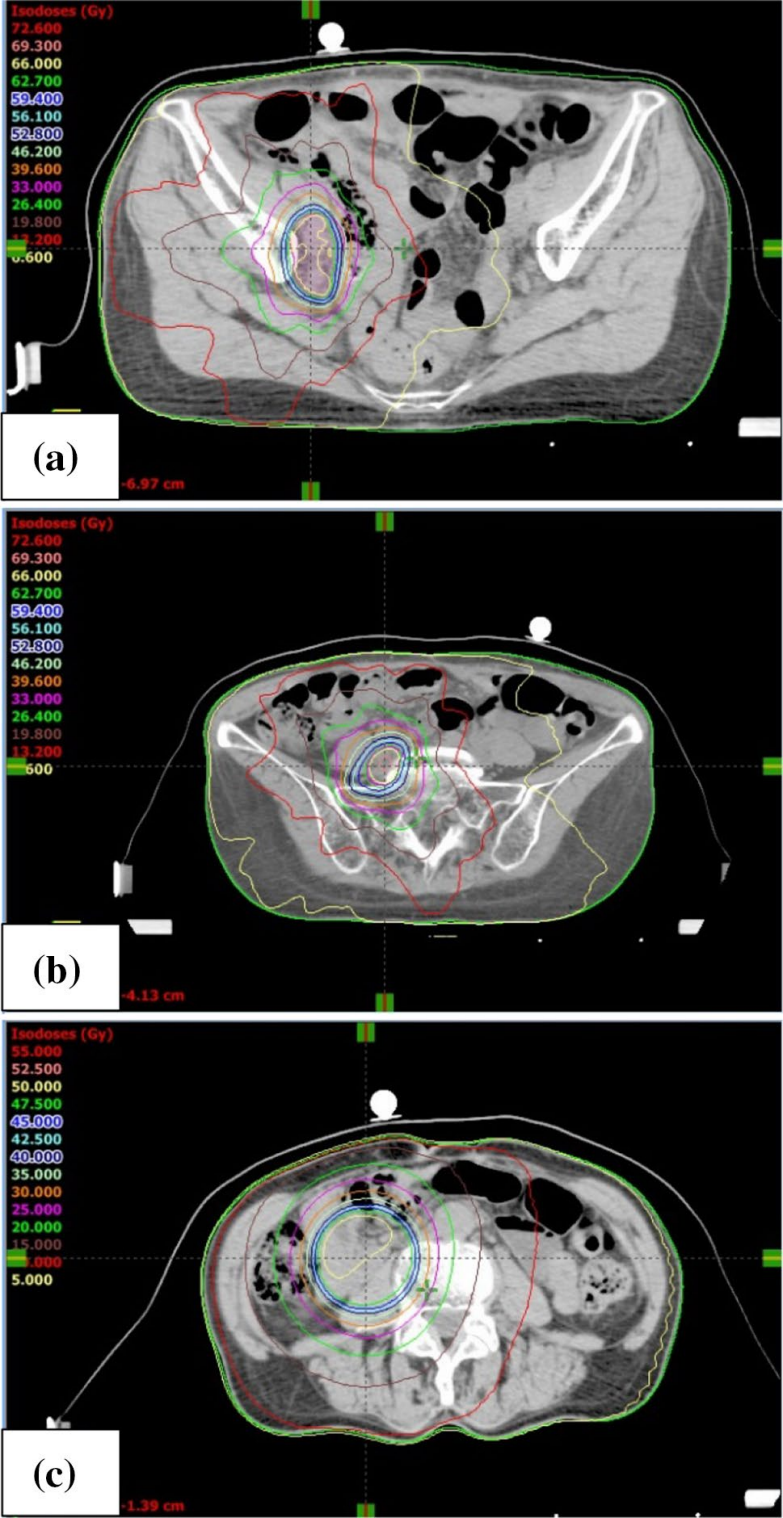
Dose distribution of IMRT. **a** Second IMRT. **b** Third IMRT. **c** Fourth IMRT. *IMRT* intensity modulated radiation therapy

A 68-year-old woman presented to our department with abnormal vaginal bleeding. Her family history was not contributory, and she had no previous medical history. Transvaginal ultrasonography revealed a tumor in the uterine cavity. On endometrial biopsy, the papillary growth of tumor cells was observed. On magnetic resonance imaging (MRI), T2-weighted images showed thickening of the endometrium and contrast enhancement (Fig. 1). On computed tomography (CT) images, no distant metastasis was observed. Transabdominal simple hysterectomy, bilateral adnexectomy, and pelvic lymphadenectomy were performed. The resected tumor filled the uterine cavity with papillary excrescence and its size was 60 mm (Fig. 2a). Histopathological examination demonstrated a papillary architecture with the papillae comprising broad fibrovascular cores and cancer had spread into the inner half of the myometrium (Fig. 2b, c). However, there were adnexal and perimetrium metastases. Based on these findings, a diagnosis of stage IIIA (pT3aN0M0) ESC was made. As postoperative adjuvant therapy, combination chemotherapy of paclitaxel and carboplatin (TC) was administered. Before the second cycle, the regimen was changed to docetaxel and cisplatin (DP) because of skin eruptions induced by paclitaxel or carboplatin. Four cycles of DP were administered. After 5 months, CT revealed tumors in the vaginal wall and left internal iliac lymph node. As fluorodeoxyglucose positron emission tomography (FDG-PET) showed accumulation with maximum standardized uptake values (SUV_max_) of 15.4 in the vaginal wall and 5.1 in the left internal iliac lymph node, the first recurrence of ESC was diagnosed (Fig. 3a, b). Concurrent chemoradiotherapy (CCRT) was performed. Chemotherapy comprised nedaplatin and docetaxel (nedaplatin 20 mg/body plus docetaxel 20 mg/body, on day two, every week for three cycles). Concurrent radiotherapy of 66 Gy (22 fractions of 3 Gy, 5 days/week) was delivered over 5 weeks using intensity modulated radiation therapy (IMRT) (Fig. 4a, b). The planning target volume (PTV) was the clinical target volume (CTV) + a 5-mm margin. Tumor regression was observed and the uptake in the recurrent site decreased considerably on the FDG-PET scan. After 4 months, the second recurrence was detected in the right internal iliac lymph node using FDG-PET with an SUV_max_ of 13.8. CCRT was performed again (Fig. 5a). The PTV was also the same. Tumor regression was observed and the uptake in the recurrent site decreased considerably on the FDG-PET scan. After 4 months, the third recurrence was detected in the right common iliac node using FDG-PET with an SUV_max_ of 9.0. CCRT was performed once more (Fig. 5b). The PTV again was the same. Tumor regression was observed and the uptake in the recurrent site decreased considerably on the FDG-PET. After 8 months, the fourth recurrence was detected in the horizontal portion of the duodenum using FDG-PET with an SUV_max_ of 8.6. IMRT (50 Gy in 25 fractions) was performed (Fig. 5c). The PTV was the same. The tumor regression was observed and the uptake in the recurrent site decreased considerably on the FDG-PET scan. After 9 months, small tumor induration was palpable on vaginal and rectal examinations. The fifth recurrence was detected in the vaginal wall, via vaginal tumor biopsy. Histological examination revealed papillary tumor cells, which were identical to those of the primary uterine lesion, with necrosis, and FDG-PET showed accumulation with an SUV_max_ of 4.2 in this site. Interstitial brachytherapy (48 Gy in 8 fractions) was performed. Tumor regression was observed and the uptake in the recurrent site decreased considerably on the FDG-PET. Grade 2 gastrointestinal fistula, nausea and radiodermatitis (CTCAE; Common Toxicity Criteria for Adverse Events, version 4.03) were observed during the treatment. During the subsequent 13-month follow-up, there has been no recurrence.

## Discussion

In this study, we report a patient with ESC with local repeated recurrence who responded to five sessions of radiotherapy to the vaginal wall-, the internal-, common-, and iliac nodes, and the horizontal portion of the duodenum. ESC is chemotherapy-resistant and has a poor prognosis [3]. Management of recurrent ESC is difficult. Radiation is the treatment of choice for women who experience recurrence at the vaginal cuff [4–6]. The histologic type is an important prognostic factor for recurrent endometrial carcinoma when radiotherapy is performed [4–7]. The present case suggested that definitive radiotherapy for locally repeated recurrent ESC was effective in situations different from the above-mentioned case of recurrence in the vaginal wall. To our knowledge, the efficacy of radiotherapy for repeated recurrent ESC has not been reported previously. A recently published study on IMRT for nodal recurrences of endometrial carcinoma demonstrated that patients who received CCRT had significantly longer median survival as compared to patients treated with radiotherapy without concurrent chemotherapy (61.9 versus 28.7 months, *p* = 0.034) [8]. Based on this information, CCRT for endometrial carcinoma with high-grade malignant potential, such as ESC, could be an effective therapy if adverse effects to adjacent organs, particularly the bowels and bladder, are acceptable. In the present case, there were three reasons for using this chemotherapy. First, it was used to enhance radiosensitization, as antitumor platinum (e.g., cisplatin and nedaplatin), paclitaxel, and docetaxel therapies have been reported as effective radiosensitizers [9–15]. Second, the chemotherapy was used for an antitumor effect, and third, nedaplatin and docetaxel were used, because they are easy to prepare and are available at outpatient clinics. Unlike conventional radiotherapy, IMRT is a method of highly conformal radiation that permits the delivery of high doses of radiation to the tumor, while minimizing doses to surrounding healthy tissues. Therefore, recently, reirradiation with IMRT has been performed. However, response time, tolerance dose, and toxic adverse effects of IMRT for recurrence are unclear. According to a report on salvage IMRT for locally recurrent nasopharyngeal cancer after definitive IMRT, the median interval between the completion of initial radiotherapy and the start of reirradiation was 27.9 (range 11.7–79.0) months; grade 3–5 toxicities occurred in 50 of the 77 patients and treatment-induced severe adverse effects were the most important contributor to mortality [16]. In another study about IMRT for paraaortic recurrence of endometrial cancer, late grade 3 or 4 gastrointestinal toxic effects occurred in 2 of 14 patients who were treated with pelvic radiotherapy as the initial treatment [17]. However, there are some points that must be kept in mind when referring to these studies. The method of setting PTV varies depending on the facility. Unlike in the present case, in the studies cited above, patients were treated with IMRT using 45–50 Gy with a 0.7–1-cm margin [17]. In the present case, adverse effects of grade 3 or greater were not observed. Thus, toxic adverse effects of IMRT may differ depending on the technique. In summary, we encountered a patient with ESC who responded to five sessions of radiotherapy. Radiotherapy (mainly IMRT) could be an effective treatment for locally repeated recurrent ESC; however, its safety should be individually considered.
